# Setting priorities for development of emerging interventions against childhood pneumonia, meningitis and influenza

**DOI:** 10.7189/jogh.02.010304

**Published:** 2012-06

**Authors:** Igor Rudan, Evropi Theodoratou, Lina Zgaga, Harish Nair, Kit Yee Chan, Mark Tomlinson, Alex Tsai, Zrinka Biloglav, Tanvir Huda, Shams El Arifeen, Mickey Chopra, Harry Campbell

**Affiliations:** 1 Centre for Population Health Sciences, The University of Edinburgh Medical School, Edinburgh, Scotland, UK; 2 Nossal Institute for Global Health, University of Melbourne, Melbourne, Australia; 3 Centre for Public Mental Health, Department of Psychology, Stellenbosch University, Stellenbosch, South Africa; 4 Centre for Public Mental Health, Department of Psychiatry and Mental Health, University of Cape Town, Cape Town, South Africa; 5 Robert Wood Johnson Health and Society Scholars Program, Harvard University, Cambridge, USA; 6 Center for Global Health, Massachusetts General Hospital, Boston, USA; 7 Andrija Štampar School of Public Health, School of Medicine, University of Zagreb, Zagreb, Croatia; 8 Child Health and Nutrition Research Initiative, International Centre for Diarrhoeal Disease Research (ICDDR,B), Dhaka, Bangladesh; 9 UNICEF, New York, USA

Acute lower respiratory infections, which broadly include pneumonia and bronchiolitis, are still the leading cause of childhood mortality. ALRI contributed to 18% of all deaths in children younger than five years of age in 2008 [1], and the main pathogens responsible for high mortality were *Streptococcus pneumoniae*, *Haemophilus influenzae* and respiratory syncytial virus [2-4]. In addition, meningitis was estimated to contribute up to 200 000 deaths each year, and influenza anywhere between 25 000 and 110 000 [1,5]. It is widely acknowledged that a major portion of this mortality should be avoidable if universal coverage of all known effective interventions could be achieved. However, some evaluations of the implementation of World Health Organization’s (WHO) Integrated Management of Childhood Illness (IMCI) strategy, which promotes improved access to a trained health provider who can administer “standard case management”, have shown somewhat disappointing results [6-8]. Only a minority of all children with life-threatening episodes of pneumonia, meningitis and influenza in developing countries have access to trained health providers and receive appropriate treatment [6-8]. Thus, novel strategies for control of pneumonia that balance investments in scaling up of existing interventions and the development of novel approaches, technologies and ideas are clearly needed.

## Emerging interventions against childhood pneumonia, meningitis and influenza

Several recent studies quantified the burden of child mortality due to childhood infections [1] and sub-divided it further according to the causing infectious pathogens [2-5]. In a series of papers that followed, we systematically reviewed the available information relevant to the emerging interventions against childhood pneumonia, meningitis and influenza [9-14]. We defined the list of emerging interventions of interest as follows: (i) the first set of emerging interventions was suggested by the officers from the Bill and Melinda Gates Foundation (BMGF) and it was based on strategic priorities that were being discussed at the Foundation in the year 2009; (ii) additional ideas were proposed by our team at the University of Edinburgh, after provisionally reviewing the literature on emerging interventions against childhood infections; (iii) the third set of emerging interventions was suggested by the 20 international experts invited to take part in the CHNRI expert panel meeting (see later). We eventually agreed to evaluate 29 emerging interventions that seemed feasible for reaching the implementation within a 10-year period (**Table 1**). We aimed to be inclusive and open-minded in their selection because some of them may still be far from implementation.

**Table 1:** The consolidated list of 29 emerging interventions against childhood pneumonia, meningitis and influenza

1	Low-cost polysaccharide conjugate vaccines for *Pneumococcus* (low-cost: US$ 3.50 per dose)
2	Low cost, cross-protective common protein vaccines for *Pneumococcus*
3	Low cost, cross-protective common protein vaccines for seasonal influenza (existing flu vaccines should be considered as a current intervention)
4	Monoclonal antibodies for passive immunization against RSV
5	Anti-RSV vaccine for use in infants
6	Anti-RSV vaccine for use in pregnant women
7	Meningitis A conjugate vaccine
8	Multivalent meningococcal vaccines
9	Combination vaccines: meningococcal + other vaccines
10	Needle-free versions of current measles vaccines
11	Heat stable versions of current measles vaccines
12	Oxygen delivery systems for low-resource settings
13	Low cost ventilatory support
14	Non-liquid pediatric antibiotic formulations for use in large scale programmes in appropriate dose
15	Vaccines against *S. aureus*
16	Passive immunization against *S. aureus*
17	Combination vaccines against multiple respiratory viruses
18	Maternal vaccination to protect neonates against neonatal sepsis: *E coli* and *Klebsiella*
19	Maternal vaccination to protect neonates against neonatal sepsis: *Streptococcus B* and *S. aureus*
20	Rapid diagnostic test for bacterial infections in children
21	Rapid multiplex assay for etiology-specific diagnosis in children
22	Rapid multiplex assay for etiology-specific diagnosis in young infants
23	Rapid diagnostic test to predict severe outcome of pneumonia episode
24	Maternal vaccination for infectious agents relevant in infants (eg, PC, Hib, influenza)
25	Effective mucosal (oral or rectal) antibiotics for neonatal infections
26	Immunomodulating agents to stimulate innate immunity
27	Surfactant replacement therapy
28	Novel interventions to reduce indoor air pollution
29	Water-free solution for hand disinfection to reduce transmission of respiratory pathogens

RSV – respiratory syncytial virus, PC – pneumococcus, Hib – *Haemophilus influenzae* Type B

## The expert opinion exercise

The CHNRI methodology for priority setting in health research (and technologies) investments was proposed as a systematic tool that can be used by those who develop research policy and/or invest in health research [15-18]. It should assist them to understand (i) the full spectrum of research investment options; (ii) the potential risks and benefits that can result from investments in different research options; and (iii) the likelihood of achieving reductions of persisting burden of disease and disability through investments in health research and health technologies. The CHNRI methodology has 3 stages: input from investors/policy-makers (who define the context and criteria for priority setting); input from technical experts (who propose, list in a systematic way, and then score different research investment options against a pre-defined set of criteria); and input from other stakeholders (weighing the criteria according to wider societal system of values). The method has been described in detail elsewhere and many examples of its implementation are publically available [19-22].

The expert opinion exercise focused only on emerging interventions and a broad, long-term (downstream) context/vision. We invited 20 leading international experts from international agencies, industry, basic science and public health research to Dubrovnik, Croatia, in September 2009. The invited experts provided opinion on how the 29 chosen emerging interventions satisfy a number of criteria relevant to prioritization of support to emerging interventions against childhood infections. Based on a modified CHNRI’s conceptual framework, 12 criteria for prioritization were developed for emerging interventions: (i) answerability (in an ethical way); (ii) low development cost; (iii) low product cost; (iv) low implementation cost; (v) likelihood of efficacy and effectiveness; (vi) likelihood of deliverability; (vii) likelihood of affordability; (viii) likelihood of sustainability; (ix) maximum potential impact on mortality burden reduction; (x) likelihood of acceptability to health workers; (xi) likelihood of acceptability to end users; (xii) predicted impact on equity. Further details about the modified CHNRI framework with the 12 criteria used for the expert panel meeting in Dubrovnik in 2009, and the process of the expert opinion exercise, are available from the corresponding author upon request.

The first task for the experts was to read the background information assembled about the 29 emerging interventions in a 285-page landscape review, later published as a series of papers [9-14]. The second task was to participate in the expert panel meeting where, over the course of 5 days and a total of 10 discussion sessions, the experts were told why each of the 12 criteria was chosen, and then they discussed how to apply them to each of the 29 emerging interventions. They were free to challenge all information provided to them in a background document and to share further personal knowledge or opinion with the group. Notes of their input were taken and the landscape review was being continuously amended. After each discussion session the experts were invited to score, independently of each other, all emerging interventions according to the 12 agreed CHNRI criteria. For each of the 29 emerging interventions and each criterion, each expert answered questions targeted to assess the likelihood of the proposed emerging interventions to comply with the priority-setting criterion. A summarized version of those questions is presented in **Table 2**. The full version of questionnaires that were used is available upon request from the corresponding author.

**Table 2:** A summarized version of questions used to assess whether proposed 29 interventions satisfy the 12 priority-setting criteria

Answerability *(“1” for Yes; “0” for No; “0.5” for Undecided)*
▪ Do we have a sufficient research and development capacity to make the intervention available on the market by 2020?
▪ Do we have a sufficient level of funding support to make the intervention available on the market by 2020?
▪ Would you say that it is likely that the remaining technical hurdles can be overcome to make the intervention available on the market by 2020?

Cost of Development (In US$) (*“1” for Yes; “0” for N; “0.5” for Undecided)*
▪ How much will it cost to get from the current stage of development to commercial availability of each emerging intervention below?
a.<US$1 billion, b.<US$ 500 million, c.<US$ 100 million

Cost of Implementation (In US$) (*“1” for Yes; “0” for N; “0.5” for Undecided)*
▪ Is it likely to be a low-cost intervention (ie, <3.50 US$ per unit?)
▪ Can we use the existing delivery mechanisms without major modifications (eg, training, infrastructure)?
▪ Is achievement of a near-universal coverage likely to be affordable to most developing countries?

Likelihood of Efficacy (*0%-100%)*
▪ Please assess the likelihood (0%-100%) that adequately powered randomized controlled trials of the interventions listed below (ROWS), conducted in developing countries, would consistently show statistically significant reduction in cause-specific mortality from each of the four causes of death listed below (COLUMNS).
a. Pneumonia, b. Meningitis, c. Neonatal sepsis, d. Influenza

Likelihood of Maximum Potential Impact on Disease Burden
▪ Please predict, for each of the 4 causes of death below (COLUMNS), the proportion of deaths in children under five years of age due to that cause that could be averted if the complete coverage with the emerging interventions listed below (ROWS) could be achieved?
a. Pneumonia, b. Meningitis, c. Neonatal sepsis, d. Influenza

Deliverability and Sustainability (*“1” for Yes; “0” for N; “0.5” for Undecided)*
▪ Taking into account (i) the infrastructure and resources required to deliver emerging interventions listed below (eg, human resources, health facilities, communication and transport infrastructure); (ii) the resources likely to be available to implement the emerging interventions at the time of introduction; (iii) overall capacity of the governments (eg, adequacy of government regulation, monitoring and enforcement; governmental intersectoral coordination), and (iv) internal and external partnership required for delivery of interventions (eg, partnership with civil society and external donor agencies), would you say that the emerging interventions would be?
a. Deliverable at the time of introduction, b. Affordable at the time of introduction, c. Sustainable for at least 10 y after the time of introduction Assessing Readiness of Health Systems to take Existing and Emerging Interventions to High Coverage Globally (90% Urban / 80% Rural) at this Point and at the Time of their Introduction (“1” – we are ready (or we will be ready); “0.5” – we may be getting closer, but are not quite ready; “0” – we will not be ready)
▪ Please study the existing and emerging interventions against childhood pneumonia, meningitis, sepsis and influenza listed below (ROWS) and the 6 “building blocks of health systems” from the WHO framework (COLUMNS). Please indicate your assessment of the level of readiness to take each of the interventions below to high coverage globally (90% urban / 80% rural) at this point in time, and following their introduction at some future point (the latter is only needed for those interventions that are NOT already available).
a. Service delivery, b. Health workforce, c. Health information systems, d. Med. products, e. Vaccines and technologies, f. Health systems financing, g. Leadership and governance

Acceptability and Equity (*“1” for Yes; “0” for N; “0.5” for Undecided)*
▪ Taking into account the overall context, intervention complexity, health workers’ behavior and the end-user population at the time of introduction,
a. Would health workers be likely to comply with implementation guidelines?, b. Would end-users be likely to fully accept the intervention?, c. Would you say that the proposed intervention has the overall potential to improve equity after 10 y following the introduction?

The process of expert assessment (scoring) of emerging interventions was performed as follows: all the experts answered the questionnaire related to each criterion by answering ‘Yes’ (1 point) or ‘No’ (0 points). They were also allowed to declare an informed but undecided answer (0.5 points) or declare themselves insufficiently informed to answer the question (missing input). Thus, the proposed research questions got a score from 20 experts for each of the 12 criteria. This score was “the proportion of maximum possible points scored when an answer was given” (ie, excluding the missing input), and it was a number between 0 and 100%. This number represented a direct measure of “collective optimism” of all the scorers toward each emerging intervention, given the criterion in question. Each of the 29 proposed emerging interventions received 12 criterion-specific scores, each ranging between 0%-100%. The criterion over which the experts were most uncertain was the cost of implementation, which was deemed very difficult to predict by most of them. We agreed that a separate exercise should be conducted in a low-income setting to improve understanding of the factors that affect this cost, and this has been done later [23].

The overall research priority score (RPS) for each intervention was computed as the mean value of 9 intermediate scores for 9 selected criteria. The reason why all 12 criteria weren’t used is because CHNRI exercise requires that the criteria need to be relatively independent of each other (similar to principal component analysis in statistics). In this exercise, we were interested in different components of the cost (development cost, product cost, implementation cost and affordability), but those 4 criteria are in fact a single criterion, and if all 4 were kept in the exercise, this would give an undue 4-fold ‘weight’ to one criterion at the expense of the others. The experts agreed that the most important of the 4 cost-related criteria related to emerging interventions is ‘development cost’, because costs of product and implementation can be met through other mechanisms (such as GAVI, PEPFAR, Global Fund, etc.). Thus, the cost of product, cost of implementation and affordability were kept out of the final score calculation. The exact scores given to all 29 emerging interventions are presented in **Table 3**. The final report on CHNRI exercise has received the approval of the experts, among whom some (mainly from the industry) wished to remain anonymous.

**Table 3:** The results of the CHNRI exercise: 29 emerging interventions with 9 intermediate scores and an overall research priority score

Rank	Emerging intervention	Answerability	Low development cost	Likelihood of efficacy	Max burden reduction potential	Deliverable	Sustainable	Acceptable to health workers	Acceptable to end users	Impact on equity	RESEARCH INVESTMENT PRIORITY SCORE
**1**	Low-cost polysaccharide conjugate vaccines for pneumococcus	0.96	0.80	0.81	0.32	0.86	0.86	1.00	0.90	1.00	**0.84**
**2**	Non-liquid pediatric antibiotic formulations for use in large-scale programs in appropriate dose	0.76	0.90	0.78	0.30	0.86	0.95	0.85	1.00	0.95	**0.82**
**3**	Low cost, cross-protective common protein vaccines for pneumococcus	0.72	0.50	0.83	0.36	0.86	0.85	1.00	0.90	1.00	**0.78**
**4**	New mucosal (oral and rectal) antibiotics for pneumonia and neonatal infections	0.58	0.70	0.60	0.22	0.80	0.90	1.00	0.94	0.89	**0.74**
**5**	Meningitis A conjugate vaccine	0.88	0.90	0.18	0.04	0.95	0.77	1.00	0.94	0.95	**0.74**
**6**	Multivalent meningococcal vaccines	0.75	0.70	0.17	0.07	0.95	0.77	1.00	1.00	0.95	**0.71**
**7**	Heat stable versions of current vaccines targeting pneumonia (eg, measles and others)	0.46	0.50	0.52	0.11	0.91	0.91	0.85	1.00	1.00	**0.69**
**8**	Needle-free versions of current vaccines targeting pneumonia (eg, measles and others)	0.57	0.50	0.49	0.10	0.86	0.91	0.85	0.95	0.95	**0.69**
**9**	Maternal vaccination for infectious agents relevant in infants (eg, PC, Hib, influenza)	0.66	0.90	0.59	0.22	0.60	0.70	0.94	0.72	0.78	**0.68**
**10**	Low cost, cross-protective common protein vaccines for seasonal flu (existing vaccines excluded)	0.61	0.50	0.52	0.15	0.82	0.75	0.90	0.80	0.90	**0.66**
**11**	Water-free solution for hand disinfection to reduce transmission of respiratory pathogens	0.88	1.00	0.69	0.18	0.65	0.50	0.67	0.56	0.67	**0.64**
**12**	Oxygen delivery systems for low-resource settings	0.81	1.00	0.77	0.21	0.65	0.55	0.65	0.70	0.44	**0.64**
**13**	Combination vaccines: meningococcal + other EPI vaccines	0.36	0.40	0.39	0.12	0.91	0.86	0.95	0.90	0.85	**0.64**
**14**	Vaccines against additional pathogens that cause pneumonia – multiple respiratory viruses	0.48	0.40	0.69	0.24	0.70	0.70	0.85	0.80	0.75	**0.62**
**15**	Anti-RSV vaccine for use in infants	0.58	0.50	0.62	0.14	0.56	0.61	0.90	0.67	0.72	**0.59**
**16**	Point-of-care diagnostic for bacterial infections in children	0.61	0.60	0.59	0.26	0.55	0.64	0.55	0.65	0.70	**0.57**
**17**	Point-of-care diagnostic for etiology-specific pathogen in young infants	0.50	0.60	0.61	0.23	0.50	0.64	0.61	0.65	0.72	**0.56**
**18**	Low cost ventilatory support	0.54	0.70	0.73	0.16	0.45	0.45	0.75	0.75	0.44	**0.55**
**19**	Anti-RSV vaccine for use in pregnant women	0.43	0.50	0.57	0.11	0.56	0.56	0.85	0.72	0.67	**0.55**
**20**	Vaccines against additional pathogens that cause pneumonia – *S. aureus*	0.47	0.60	0.40	0.12	0.64	0.55	0.85	0.75	0.55	**0.55**
**21**	Point-of-care diagnostic to distinguish viral and bacterial infections in young infants	0.36	0.60	0.61	0.20	0.50	0.64	0.61	0.65	0.72	**0.54**
**22**	Point-of-care diagnostic to predict severe outcome of pneumonia episode	0.29	0.40	0.63	0.32	0.41	0.59	0.67	0.85	0.72	**0.54**
**23**	Novel interventions to reduce indoor air pollution	0.64	0.90	0.54	0.12	0.50	0.40	0.42	0.61	0.56	**0.52**
**24**	Immunomodulating agents to stimulate innate immunity	0.51	0.50	0.43	0.10	0.38	0.38	0.75	0.81	0.50	**0.48**
**25**	Monoclonal antibodies for passive immunization against RSV	0.71	0.90	0.63	0.09	0.17	0.17	0.65	0.56	0.33	**0.47**
**26**	Maternal vaccination to protect neonates against major causes of neonatal sepsis – *Streptococcus B*, *Staphylocossus*	0.25	0.50	0.20	0.07	0.45	0.50	0.85	0.75	0.55	**0.46**
**27**	Surfactant replacement therapy	0.62	0.80	0.41	0.08	0.33	0.19	0.63	0.69	0.38	**0.46**
**28**	Maternal vaccination to protect neonates against major causes of neonatal sepsis – *E coli*, *Klebsiela*	0.25	0.40	0.25	0.05	0.45	0.50	0.85	0.70	0.50	**0.44**
**29**	Passive immunization against *Staphylococcus*	0.58	0.60	0.32	0.07	0.33	0.33	0.65	0.72	0.28	**0.43**

RSV – respiratory syncytial virus, PC – pneumococcus, Hib – *Haemophilus influenzae* Type B

**Figure:**
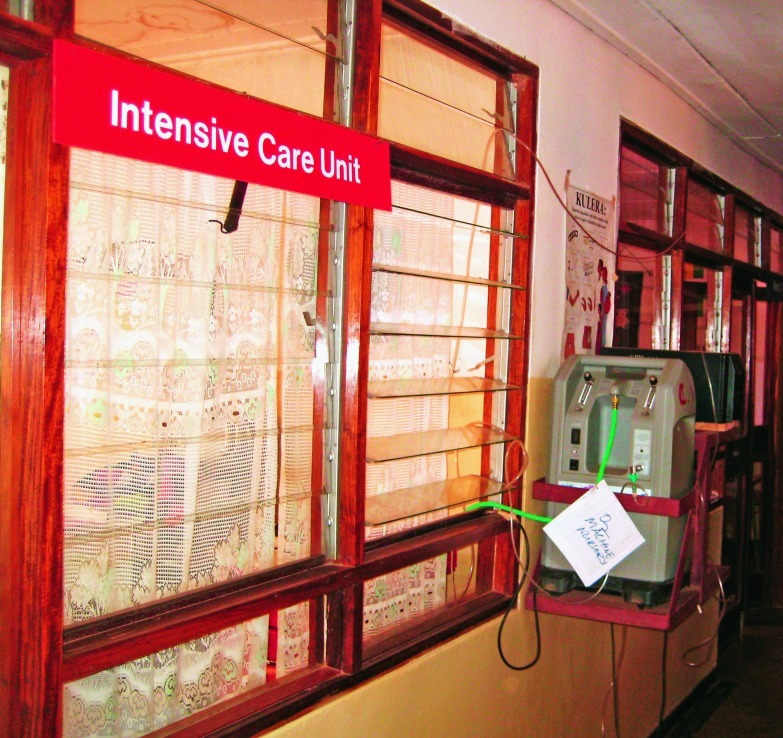
Photo: Courtesy of Alasdair Campbell, private collection

## The main messages

**Table 3** shows that the experts declared most of their collective optimism to improvement of low-cost pneumococcal conjugate vaccines. This was followed by the development of non-liquid and mucosal antibiotic pediatric formulations with improved deliverability and acceptability in low resource settings. The development of common-protein pneumococcal vaccines and multivalent meningococcal vaccines were seen as the third most promising emerging intervention. Following this cluster at the top, the second level of priority was assigned to improvements in existing vaccines (eg, measles or *H. influenzae type b*) to enable needle-free delivery and heat stability. Similar overall scores were given to evaluations of maternal immunization, improved use of oxygen systems and the development of combination vaccines and vaccines against major viral pathogens. The next level of priority was assigned to various diagnostic tools, the impact of which is currently limited with sub-optimal levels of access to care, care-seeking behavior and the availability of 1st and 2nd line antibiotics. Interventions that proposed passive immunization, action on risk factors such as indoor air pollution or poor sanitation, or development of vaccines against sepsis-causing bacterial pathogens such as *S. aureus* or *E coli* received the lowest scores (**Table 3**).

An extended version of the results of the CHNRI process with the current status of each emerging interventions’ development, the key challenges that remain to be addressed, the visual representation of scores given by the expert panel to each intervention and the assessment of potential effectiveness of each intervention is available in the series of papers published elsewhere [9-14]. It should be noted that the assessment of potential effectiveness (**Table 3**) can also range from 0%-100%, but its interpretation is different than of the other 11 criteria; rather than measuring collective optimism, it actually predicts the proportion of mortality burden that could be averted through implementation.

Pneumococcal conjugate vaccines, which were treated as emerging interventions back in 2009 because of a very low uptake in low and middle income countries at the time, achieved scores over 80% for all criteria apart from “low product cost” – which indeed ended up being the main point of discussion once they were introduced. In comparison, common protein pneumococcal vaccines are still held back by concerns over answerability (although it is getting closer to 80%), and over all criteria related to their future cost. Other interventions show quite different score profiles. For example, anti-RSV vaccine for use in infants failed on all criteria apart from “acceptance for health workers”, whereas monoclonal antibodies for passive immunization against RSV failed entirely on product cost, affordability and sustainability concerns, although product development cost was considered feasible. The introduction of oxygen systems was considered answerable and did not suffer from major cost concerns, but these systems were not deemed sustainable, sufficiently acceptable and equitable. In comparison, common protein flu vaccines were considered sustainable, acceptable and equitable, but there were still concerns about answerability and costs of development and of the final product.

## Conclusion

In accordance with other similar exercises with CHNRI methodology the process showed some clear advantages. The context and the criteria were transparent and the management of the process was overseen by the funding agency (BMGF) over its entire duration. This kind of partnership should result in better understanding and promote ownership and commitment to the main messages of the expert opinion exercise. The scoring process was highly systematic and structured. It was free from undue influence from prominent members within the expert group, because all the experts submitted their opinions and scores independently from each other. The varied mix of the experts from different backgrounds ensured that the scientific assessment of the research priorities is combined with a view of the broader community in which the priorities would be implemented. The entire process from the initial to the final stages was documented and can be viewed and challenged at any point in time. The final result of the process was a simple quantitative outcome (“research priority score”), which measured the “value” of each research option when all the criteria and views were taken into account. This “value” can be combined with the predicted cost of further research and development needs in order to derive an optimal mix of emerging interventions to be funded from a limited budget.
